# Influences and mechanism of erythropoietin on the cognitive function of vascular dementia rats

**DOI:** 10.18632/aging.205178

**Published:** 2023-11-06

**Authors:** Zhipeng Tang, Xiuqin Li, Nan Yin, Ming Zhao, Qingchuan Hu, Peiyuan Lv

**Affiliations:** 1 Department of Neurology, Hebei Medical University, Shijiazhuang, Hebei, China; 2 Department of Neurology, Hebei General Hospital, Shijiazhuang, Hebei, China; 3 Department of Geriatric Medicine, Hebei General Hospital, Shijiazhuang, Hebei, China; 4 Department of Laboratory, Hebei General Hospital, Shijiazhuang, Hebei, China; 5 Hebei Provincial Key Laboratory of Cerebral Networks and Cognitive Disorders, Shijiazhuang, Hebei, China

**Keywords:** vascular dementia, erythropoietin, oxidative stress, cell apoptosis

## Abstract

Purpose: To investigate the influences and mechanism of erythropoietin (EPO) on the cognitive function of vascular dementia (VD) rats.

Methods: 1) Spatial memory capacity was assessed by Morris water maze test; 2) Pathological conditions of brain tissues were detected by hematoxylin-eosin (HE) staining; 3) The effect of treatment on apoptosis was observed by terminal deoxynucleotidyl transferase-mediated dUTP nick end labeling (TUNEL) staining; 4) Western blotting was used to examine the protein expression in hippocampal neurons.

Results: The escape latency and swimming distance in the EPO group were much shorter than those in the Model group on the fifth day. In the spatial exploration test, the time spent in the target quadrant was longer, the number of platform crossings was larger and the swimming speed was higher in the Sham group and EPO group than those in the Model group. The results of HE staining showed that the cells in the hippocampal CA1 region were arranged closely in the Sham group, loosely and disorderly in the Model group but significantly better in the EPO group. Compared with that in the Model group, the number of apoptotic cells in the EPO group was obviously smaller. The results of Western blotting revealed that the expressions of EPO, p-EPOR, p-SHP2, p-TrKB, p-PI3K, p-ERK1/2 and Bcl-2 rose, while the expressions of P22, P47, Caspase-3, Caspase-9 and Bax significantly declined in the EPO group.

Conclusions: EPO can effectively ameliorate the cognitive dysfunction induced by chronic hypoperfusion in VD rats by mediating oxidative stress-related pathways.

## INTRODUCTION

Vascular dementia (VD), a severe cognitive dysfunction syndrome, is defined as an ischemic or hemorrhagic cerebrovascular disease induced by chronic hypoperfusion under ischemia and hypoxia [1, 2]. Thought slowness, forgetfulness, depression and anxiety, disorientation and loss of executive function are the symptoms of VD. VD accounts for approximately 17-20% of all types of dementia, making it the second leading form of dementia after Alzheimer's disease, which frequently occurs in the elderly [3, 4].

Hippocampus plays an impressive role in related cognitive function and the important brain area can directly participate in the storage and processing of information [5]. As the advanced center of memory and learning, hippocampus is also the sensitive area of ischemia and hypoxia. In the case of long-term chronic hypoperfusion, ischemia and hypoxia can easily cause the loss and necrosis of hippocampal neurons, especially in the CA1 region, memory loss, and emotional or personality changes [6].

Oxidative stress, as a main risk factor for the occurrence and development of VD, can lead to an imbalance of antioxidant active substances and reactive oxygen species (ROS), causing neuronal damage. Erythropoietin (EPO), a hypoxia-induced growth factor of the mammalian kidney, is named for its role in hematopoiesis. The function of the hippocampus is heavily dependent on adequate energy supply, and is highly susceptible to hypoxia. Long-term or repeated hypoxia can cause neuronal damage in the hippocampus, resulting in progressive inflammation, cell death and degenerative changes [7, 8]. In the case of hypoxia, hypoxia-inducible factor (HIF) will induce the production of endogenous EPO and its receptor (EPOR) in the brain. EPO exerts its neuroprotective effect by activating its receptor and blocking the apoptosis pathway to reduce stress-induced injury. Although EPOR can be highly expressed in the hypoxic-ischemic brain, insufficient production of endogenous EPO in the brain may be the cause of loss of specific neuronal populations [9]. With the ability to regulate erythropoiesis, promote the proliferation and differentiation of bone marrow hematopoietic stem cells and treat anemia caused by various reasons, EPO has been widely applied in clinic. Recently, numerous studies have shown that functional EPO and EPOR can be synthesized by astrocytes and neurons in the brain, and exert a protective effect against neuronal injury [10, 11]. Therefore, it is believed that EPO of the central nervous system is an endogenous system to prevent neurodegeneration. Previous studies found that if the EPO/EPOR system is upregulated in neuronal injury, the concentration of EPO in cerebrospinal fluid (CSF) will increase. However, few published data are available on the changes in the EPO level in CSF of patients with vascular cognitive impairment up to now.

Therefore, this study aims to investigate the influence of EPO on VD rats and its mechanism, thereby providing a new therapeutic target for VD.

## RESULTS

### EPO could alter the spatial learning and memory capacity of VD rats

Morris water maze test, a classical method to assess the spatial learning and memory capacity of rodents, can objectively reflect the changes in spatial memory capacity of animals, which has been widely used in the evaluation of learning capacity, neurology, intelligence, neuropsychology, behavior and other disciplines.

**Figure 1:**
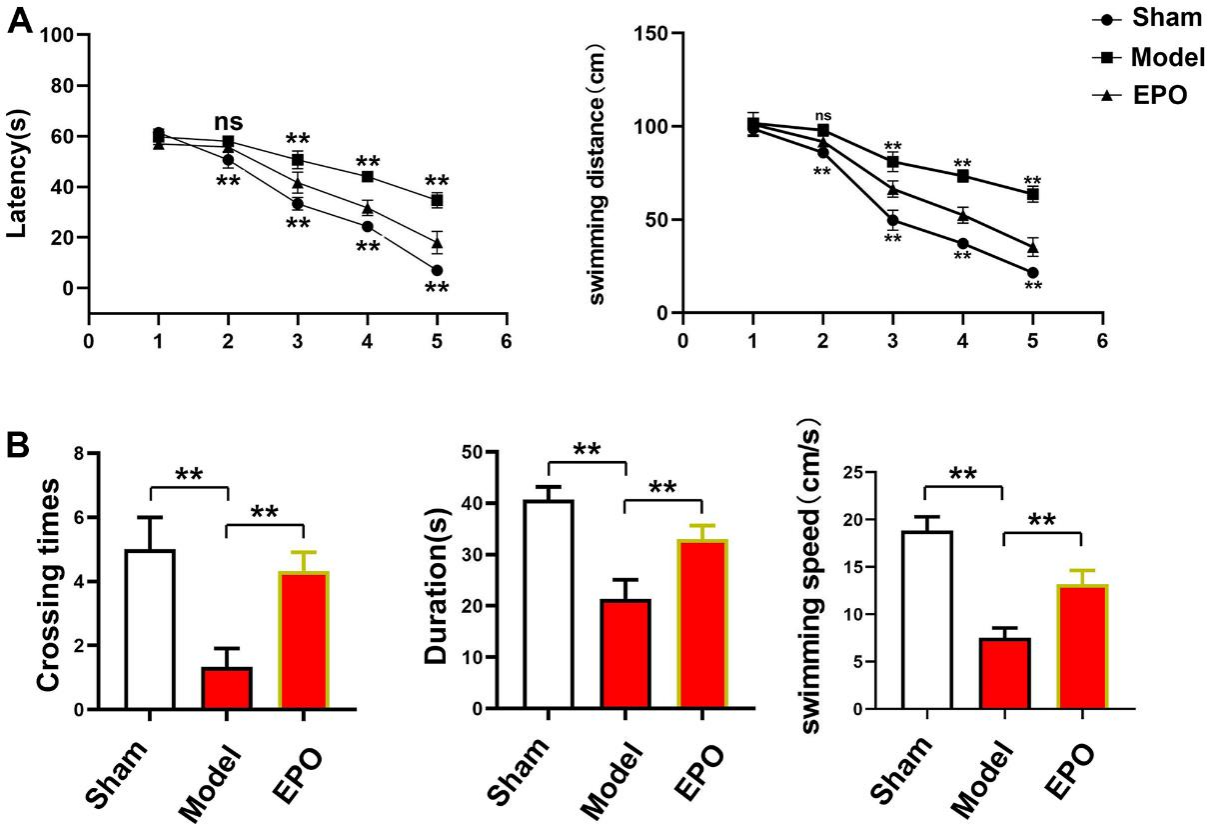
**Changes in escape latency, number of platform crossings and time spent in the target quadrant in the three groups.** (**A**) Changes in escape latency and swimming distance from the first to the fifth days in each group; (**B**) The number of platform crossings, time spent in the target quadrant and swimming speed. ^**^P<0.01; ^ns^ P>0.05.

The escape latency was gradually shortened in each group with time, and there were significant differences among the three groups (P<0.05). On the first day, there were no significant differences in the escape latency and swimming distance among the three groups (P<0.05). On the 2nd day, the escape latency and swimming distance in the Sham group and EPO group were much shorter than those in the Model group, with significant differences (P<0.05). From the 3rd to 5th day, the escape latency and swimming distances in the EPO group and Sham group were also significantly shorter than those in the Model group, and there were statistically significant differences (P<0.05), but the EPO group and Sham group had no statistically significant differences at different time points (P>0.05). Besides, the number of platform crossings was larger and the swimming speed was higher in the Sham group and EPO group than those in the Model group (Sham vs. Model: P<0.05; EPO vs. Model: P<0.05). Similarly, the time spent in the target quadrant in the Sham group and EPO group was significantly longer than that in the Model group (Sham vs. Model: P<0.05; EPO vs. Model: P<0.05) (Figure 1).

### Morphological changes in neurons in hippocampal CA1 region of VD rats after EPO administration

**Figure 2:**
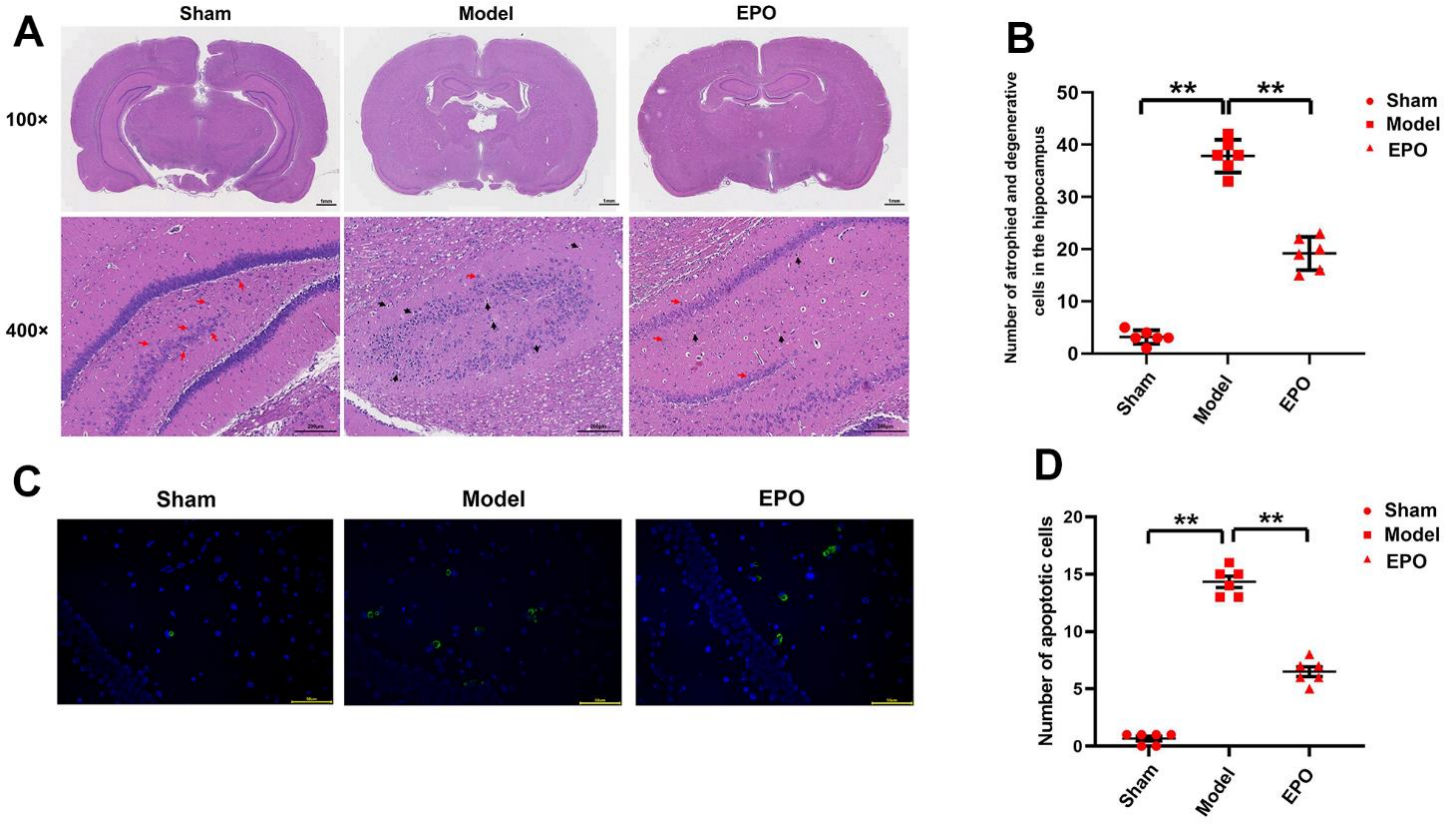
**Morphological changes in hippocampal CA1 region in the three groups.** (**A**) Morphological changes in hippocampal CA1 region in the three groups (100×/400×); (**B**) The number of degenerated cells in the hippocampus; (**C**) Diagram of TUNEL assay results; (**D**) The number of apoptotic cells in hippocampus of the three groups. ^**^P<0.01.

Microscopically (100×), in the Sham group, the number of pyramidal cells in hippocampal CA1 region was larger and the cells were arranged closely; in the Model group, the pyramidal cells in hippocampal CA1 region were arranged loosely and the number of cells declined; in the EPO group, the number and density of pyramidal cells in hippocampal CA1 region were larger than those in the Model group and similar to those in the Sham group. Microscopically (400×), in the Sham group, the pyramidal cells in hippocampal CA1 region were closely arranged, uniform in size, intact in structure, stained dark in the cytoplasm, regular in shape, and clear in the nucleolus. In the Model group, the pyramidal cells were arranged loosely and disorderly with different sizes as well as slight cytoplasmic staining, with pyknosis and deep staining of nuclei, and vacuole disappearance of nucleoli in some cells. In the EPO group, the above conditions were significantly better than those in the Model group (P<0.05). Furthermore, there were almost no apoptotic cells in the Sham group, while a larger number of apoptotic cells were microscopically observed in the Model group than the EPO group (Figure 2).

### Influences of EPO on the protein expressions of EPO, p-EPOR, P22, P47 and p-SHP2 in hippocampal neurons of VD rats

**Figure 3:**
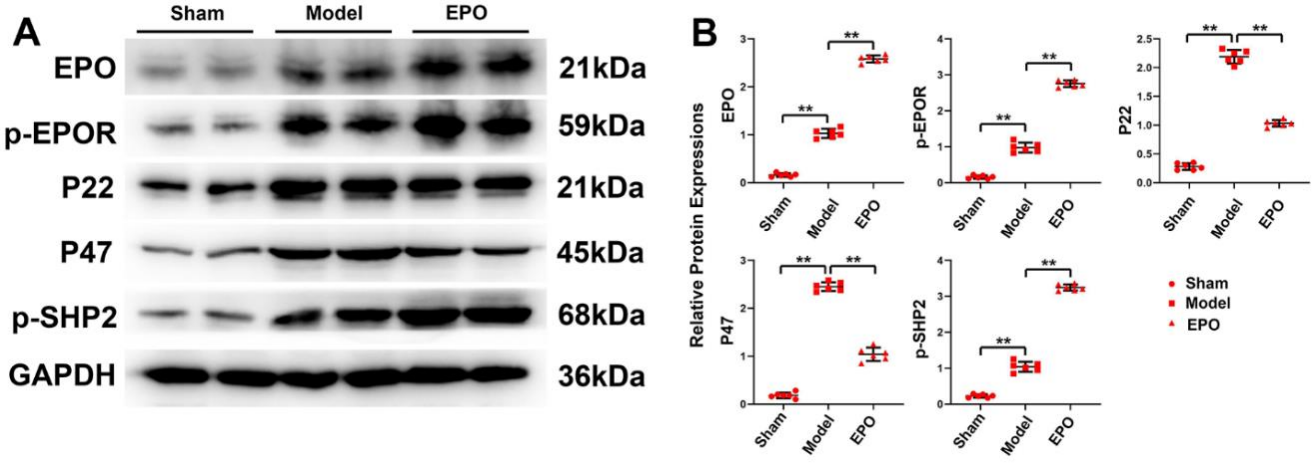
**Protein expressions of EPO, p-EPOR, P22, P47 and p-SHP2 in the three groups.** (**A**) Representative protein bands of EPO, p-EPOR, P22, P47 and p-SHP2 in hippocampus of the three groups. (**B**) Relative protein expressions of EPO, p-EPOR, P22, P47 and p-SHP-2 in hippocampus of the three groups. ^**^P<0.01.

The results of Western blotting revealed that the protein expressions of EPO, p-EPOR, P22, P47 and p-SHP2 in the Sham group were significantly lower than those in the Model group (P<0.05), with statistically significant differences. The physiological effect of EPO combined with EPOR is very weak, and long-term hypoxia can stimulate the expression and phosphorylation of EPOR in pyramidal cells, consequently activating the downstream signal pathway and improving the adaptability of rats to environmental changes. With the addition of exogenous EPO, the protein expressions of EPO, p-EPOR and p-SHP-2 increased significantly (P<0.05), while the relative protein expressions of P22 and P47 decreased significantly (P<0.05). It can be seen that exogenous EPO can further promote the expression and phosphorylation of EPOR, thus inhibiting oxidative stress (Figure 3).

### Influences of EPO on the expressions of apoptosis-related proteins in hippocampal neurons of VD rats

**Figure 4:**
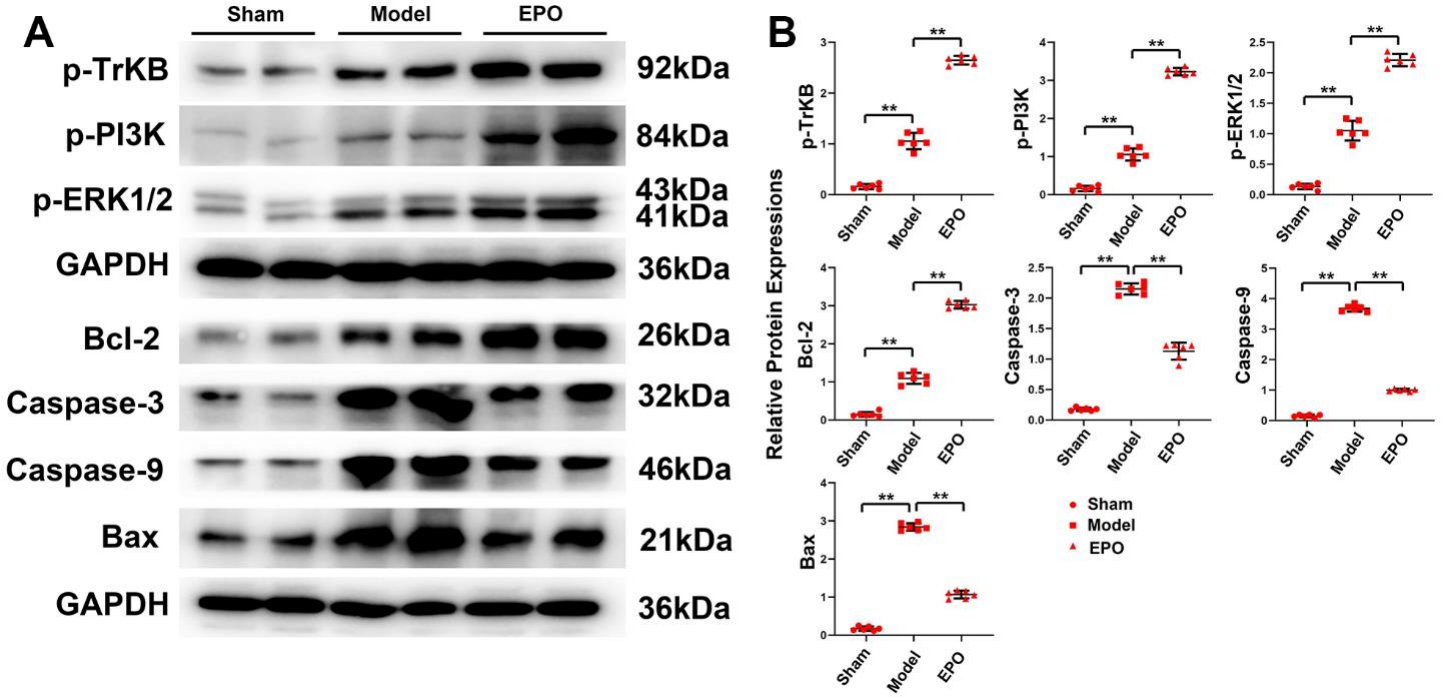
**Expressions of BDNF/TrKB/PI3K/ERK1/2 axis and apoptosis-related proteins, and apoptosis of hippocampal neurons in the three groups.** (**A**) Representative protein bands of p-TrKB, p-PI3K, p-ERK1/2, Bcl-2, Bax, Caspase-9 and Caspase-3 in hippocampus of the three groups; (**B**) Relative protein expressions of p-TrKB, p-PI3K, p-ERK1/2, Bcl-2, Bax, Caspase-9, and Caspase-3 in hippocampus of the three groups; ^**^P<0.01.

Significantly higher protein expressions of p-TrKB, p-PI3K, p-ERK1/2, Bcl-2, Bax, Caspase 3 and Caspase 9 were found in the Model group than those in the Sham group (P<0.05). Compared with those in the Model group, the protein expressions of p-TrKB, p-PI3K, p-ERK1/2 and Bcl-2 increased, while those of BAX, Caspase-9 and Caspase-3 significantly decreased in the EPO group (P<0.05). To sum up, EPO can significantly inhibit the excessive apoptosis of hippocampal neurons under chronic hypoperfusion (Figure 4).

### Influences of EPO on the expressions of p-EPOR, P22, p-SHP2, p-TrKB, p-PI3K, p-ERK1/2, Bcl-2 and Bax in hippocampal neurons *in vitro*

**Figure 5:**
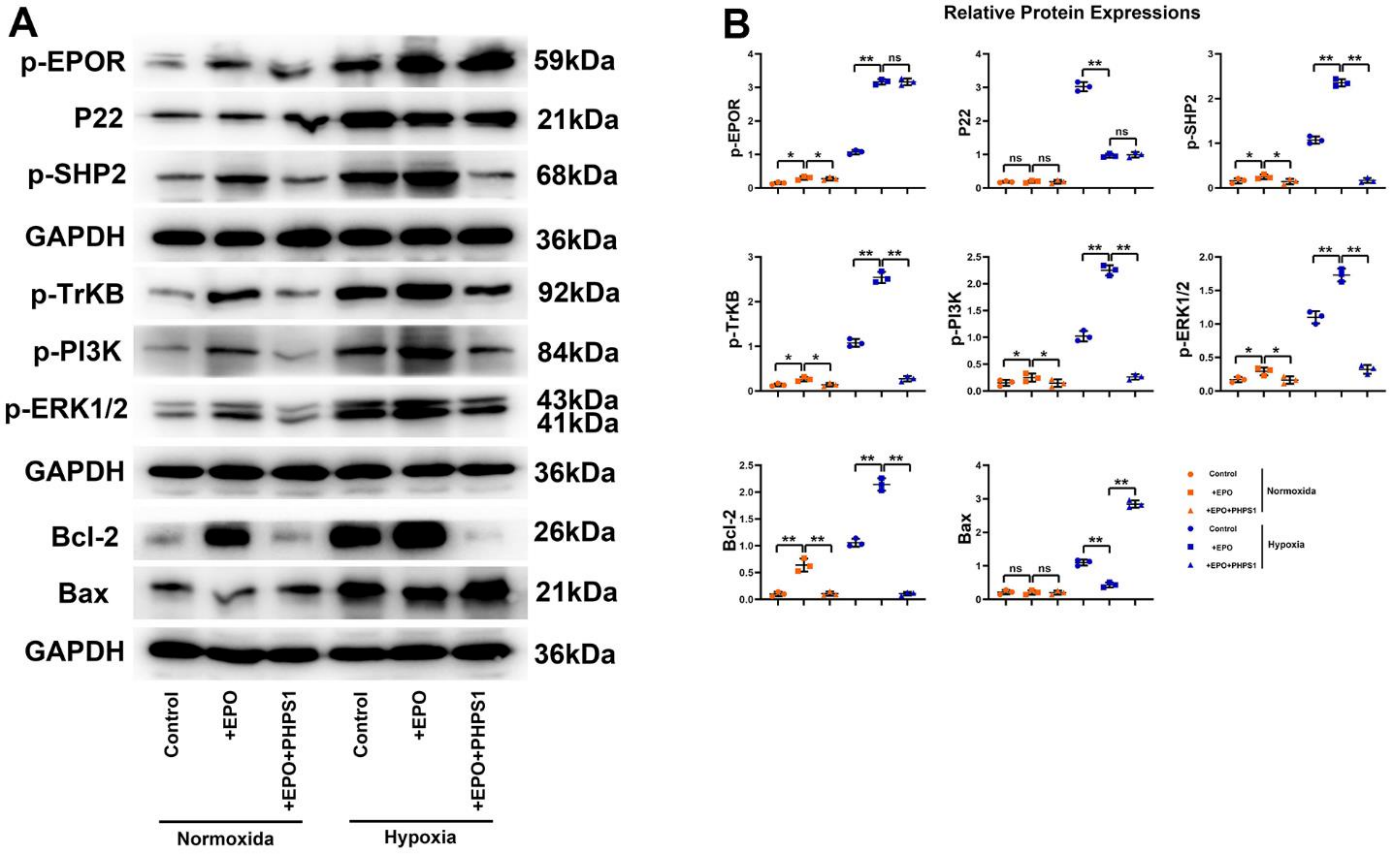
**Protein expressions of p-EPOR, P22, p-SHP2, p-TrKB, p-PI3K, p-ERK1/2, Bcl-2 and Bax in hippocampal neurons *in vitro*.** (**A**) Representative protein bands of p-EPOR, P22, p-SHP2, p-TrKB, p-PI3K, p-ERK1/2, Bcl-2 and Bax in hippocampus *in vitro*; (**B**) Relative protein expressions of p-EPOR, P22, p-SHP2, p-TrKB, p-PI3K, p-ERK1/2, Bcl-2 and Bax in hippocampus *in vitro*. ^**^P<0.01; ^*^P>0.05; ^ns^ P>0.05.

**Figure 6:**
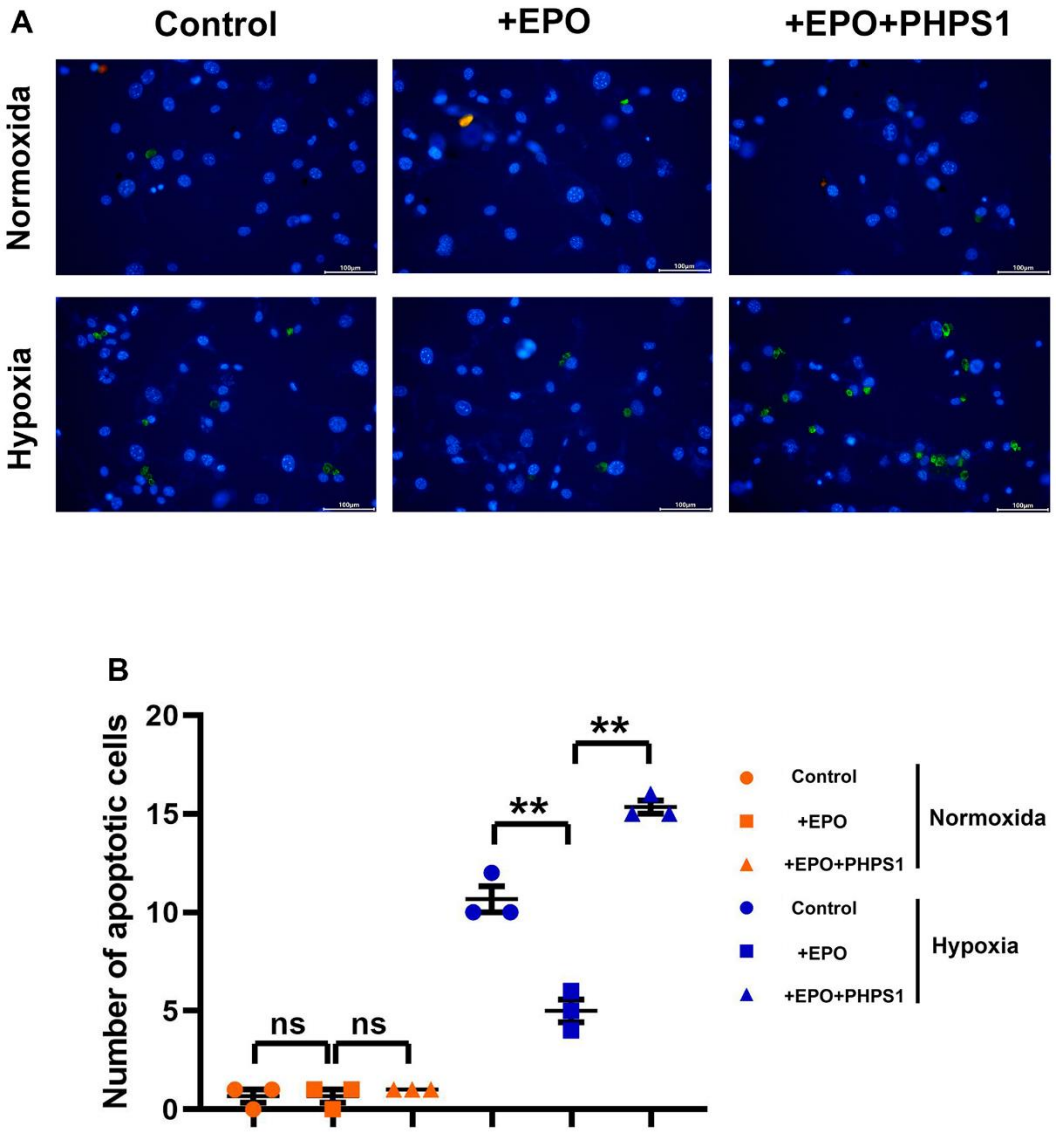
**Under hypoxic conditions, EPO can inhibit hippocampal neuronal apoptosis by activating SHP2.** (**A**) Diagram of TUNEL assay results; (**B**) The number of apoptotic cells in hippocampus of in each group. ^**^P<0.01; ^ns^ P>0.05.

**Figure 7:**
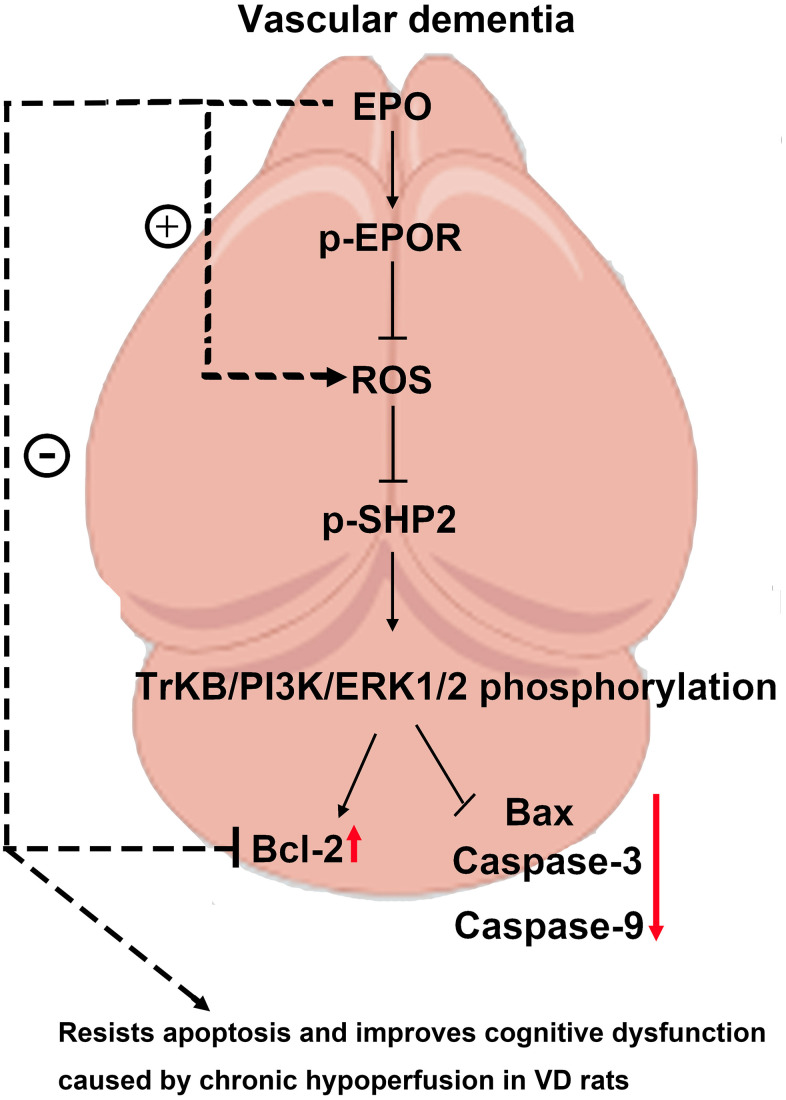
EPO can regulate the oxidative stress in hippocampal neurons, thereby resisting apoptosis and inhibiting inflammatory response through the BDNF/TrKB/PI3K/ERK1/2 axis to effectively ameliorate the cognitive dysfunction caused by chronic hypoperfusion in VD rats.

The results of Western blotting showed that the protein expressions of P22 and Bax in Control group, EPO group and EPO+PHPS1 group were not significantly different under normoxia (P>0.05). The EPO group had higher protein expressions of p-EPOR, p-SHP2, p-TrKB, p-PI3K, p-ERK1/2 and Bcl-2 than the Control group and the EPO+PHPS1 group. Under hypoxia, however, the protein expressions of p-EPOR, p-SHP-2, p-TrKB, p-PI3K, p-ERK1/2 and Bcl-2 in the EPO group were significantly higher (P<0.05), while the protein expressions of P22 and Bax were significantly lower than those in the Control group (P<0.05). Compared with the EPO group, the EPO+PHPS1 group had significantly lower protein expressions of p-SHP2, p-TrKB, p-PI3K, p-ERK1/2 and Bcl-2 (P <0.05), a higher expression of Bax (P<0.05), and the same expressions of p-EPOR and P22 (P>0.05) (Figure 5). And TUNEL results showed that the number of apoptosis in each group was very small and there was no significant difference in the normoxic condition, while in the hypoxia situation, the number of apoptosis in the EPO group was significantly reduced compared with the control group, and the number of apoptosis in the EPO+PHPS1 group was significantly increased (Figure 6). It can be inferred that EPO can regulate the oxidative stress in hippocampal neurons, thereby resisting apoptosis and inhibiting inflammatory response through the BDNF/TrKB/PI3K/ERK1/2 axis to effectively ameliorate the cognitive dysfunction caused by chronic hypoperfusion in VD rats (Figure 7).

## CONCLUSIONS

VD (a clinical syndrome of learning and memory decline and cognitive dysfunction caused by cerebral ischemia and hypoxia in various cerebrovascular diseases) belongs to an age-related neurodegenerative disease, which is the second major type of dementia after Alzheimer's disease, accounting for about 20% of the total [12, 13]. With the aging of the population, the incidence of VD is rising, seriously threatening the physical and mental health of the elderly and bringing serious burden to society and family. However, there is still a lack of effective treatment drugs for VD in clinic [14]. Therefore, it is urgent to explore the pathogenesis of VD and develop new preventive and therapeutic methods.

EPO is a glycoprotein of the type I cytokine superfamily, which can bind to the surface receptor of erythroid progenitor cells to enhance the proliferation, differentiation and maturation of erythrocytes. In recent years, EPO has been found to have a wide range of biological activities in addition to its role in promoting erythropoiesis, especially in the protection of the nervous system [15]. Recent studies have found that EPO and its receptor can be expressed in various parts of the brain such as central neurons and vascular endothelial cells of mammals. When exposed to hypoxia, trauma and other stress stimuli, the expression of endogenous EPO and its receptor will be up-regulated, which plays an impressive role in neuroprotection through a variety of mechanisms [16]. Many studies have found that EPO can protect neurons by inhibiting inflammatory responses, scavenging oxygen free radicals, reducing neuronal apoptosis, promoting angiogenesis and improving energy metabolism, increasing the transformation and migration of neuronal precursors, enhancing synaptic plasticity, and protecting blood-brain barrier [17]. In this study, whether EPO has the same neuroprotective effect on patients with VD, and whether EPO has potential application value in the prevention or treatment of VD were investigated. In this study, the results of Morris water maze test showed that after the addition of EPO, the escape latency and swimming distance were significantly shortened, and the time spent in the target quadrant, the number of platform crossings and swimming speed were all significantly increased. As observed by HE staining, EPO could improve the pathological changes of VD rats, suggesting that EPO can ameliorate the cognitive dysfunction of VD rats, consistent with other research results.

Apoptosis is a self-destructive mechanism in response to adverse environmental stimuli, involving the activation, expression and regulation of a series of genes. Both under- and over-regulation of apoptosis can cause human diseases. Chronic hypoperfusion-induced hippocampal neuronal injury involves the interaction of many factors including the increase of excitatory amino acid concentration in synaptic cleft, the massive production of free radicals, intracellular calcium overload, the release of inflammatory factors, the imbalance of pro-apoptotic and anti-apoptotic proteins, etc., which eventually lead to the apoptosis of a large number of neurons [18, 19]. HIF-1α induces the expression of EPO and its receptor in the brain under hypoxic conditions. EPO binding to its receptor can block apoptosis and thus reduce neuronal injury induced by different types of stress. SHP2 activation can activate TrKB (a receptor of BDNF) in cortical neurons. BDNF/TrKB/PI3K/ERK1/2 axis is a key signaling pathway involved in cell proliferation, survival, differentiation and apoptosis [20]. Activation of this pathway can regulate the phosphorylation of downstream substrates and exert a wide range of biological effects, such as anti-apoptosis and cell survival. Thus, it is hypothesized that EPO may affect SHP2 activation and then mediate the BDNF/TrKB/PI3K/ERK1/2 axis, thus affecting apoptosis and inflammatory response.

In this study, the protein expressions of p-EPOR, p-TrKB, p-PI3K, p-ERK1/2, and Bcl-2 increased, while the protein expressions of P22, P47, Bax, Caspase-9, and Caspase-3 decreased significantly in the EPO group. The results of TUNEL assay showed that EPO could reduce the number of apoptotic cells in VD rats. The above findings were then verified by *in vitro* tests.

In conclusion, EPO can regulate the oxidative stress in hippocampal neurons, thereby resisting apoptosis and inhibiting inflammatory response through the BDNF/TrKB/PI3K/ERK1/2 axis to effectively ameliorate the cognitive dysfunction caused by chronic hypoperfusion in VD rats, which provides a new therapeutic target for VD.

## MATERIALS AND METHODS

### Laboratory animals

Thirty male Wistar rats (four months old and 250ght g, from the Laboratory Animal Center of Hebei Medical University) were adaptively fed in the Laboratory Animal Center for one week before experiments, during which the room temperature was maintained at 23he° C, and the humidity was maintained at 40%-60%, with a 12/12 h light/dark cycle. During feeding, the rats had free access to food and water. Besides, the investigators adhered strictly to the guidelines for ethics and use of laboratory animals.

### Animal modeling and grouping

Chronic cerebral ischemia was induced by bilateral common carotid artery ligation to alter the pathophysiology in hippocampal neurons, specifically as follows: a) The rats were anesthetized by intraperitoneal injection of 3% sodium pentobarbital (30 mg/kg); the mid-neck hair was removed and the rats were disinfected; a 1.5 cm-long incision was made along the midline of the neck; the subcutaneous tissue was dissected bluntly; bilateral common carotid arteries were exposed and gently dissected from the carotid sheath and vagus nerve; b) Bilateral common carotid artery ligation was performed with 4-0 silk thread and cut off in the middle; the heartbeat and respiration status of rats were observed; the anatomical structure of subcutaneous tissue without abnormality was restored; the incision was sutured with #0 surgical thread and the local skin was disinfected with iodophor. After the operation, each rat was placed in a separate cage and the wound healing and abnormal death were observed. In the Sham group, bilateral common carotid arteries were only isolated with the silk loop, without ligation and cutting. Forty rats were randomly divided into 3 groups: 1) Sham group (n=10): normal saline given by gavage, once a day; 2) Model group (n=10): normal saline given by gavage, once a day; 3) EPO group (n=10): EPO 5000 UI/kg, once a day. The agents were orally given at 10:00-12:00.

### Morris water maze test

The Morris water maze test included positioning navigation test on the first five days and space exploration test on the sixth day. In the positioning navigation test, the rats were gently put into the water facing any quadrant of the pool wall and allowed to swim for 120 s to find the hidden platform. If the rat successfully found the platform in the given time, it could rest for 20 s on the platform; otherwise, the investigator would help the rat to stay on the platform for 20 s. Once the rat fell off the platform or jumped into the water within 20 s, it was put back on the platform to ensure that the time interval reached 20 s, so that the rat had the same amount of time to get the spatial information. After each training, the rat was dried with dry towels in time to avoid the influence of hypothermia. Then the platform was removed and the space exploration test was carried out, as follows: The rat was put into the water at the same place in any quadrant, and the swimming trajectory within 120 s, the time spent in the target quadrant of the hidden platform and the number of platform crossings (the location of the hidden platform was marked by the blue bot on the computer) were recorded.

### Hematoxylin-eosin (HE) staining

The tissues were sectioned and deparaffinized with hematoxylin in aqueous solution for several min, followed by color separation in acid water and ammonia water for several seconds, respectively. After rinsing with running water for 1 hour, the sections were placed in distilled water for a moment, dehydrated with alcohol for 10 min, stained with alcohol eosin for 3 min, dehydrated with pure alcohol, transparentized with xylene, and mounted with neutral gum. Finally, the morphological changes in hippocampal neurons were observed under a light microscope (100i and 400i) (Leica DM750, Japan).

### Terminal deoxynucleotidyl transferase-mediated dUTP nick end labeling (TUNEL) staining

After pretreatment with polylysine, the sections were deparaffinized into water, digested with Proteinase K freshly diluted at 1:200 by 0.01 M TBS at 37° C for 10 min, washed 3 times with TBS (2 min each time) and then moistened with 20 μL of labeling buffer containing 1 μL each of TdT and BIO-d-UTP. After the excess liquid on the section was discarded, the section was placed in a wet box at 37° C for 2 hours and washed with 0.01 M TBS for 3 times (2 min each time). Then each section was added with 50 μL of blocking buffer, and placed at room temperature for 30 min. In addition, 10 μL of SABC was mixed with 1 mL of SABC diluent and 50 μL of the mixture was added to each section. After 30 min of reaction and 4 times of washing in 0.01 M TBS (5 min each time), the sections were counterstained with DAPI staining solution and washed with distilled water. Under a fluorescence microscope, the green granules in the nucleus indicated positive cells, that is, apoptotic cells, while the blue granules indicated negative cells.

### Cell processing

One-day-old rats were disinfected with 75% alcohol, and the brain tissues were harvested, washed with PBS once and quickly placed into pre-chilled high-glucose DMEM. Then the bilateral hippocampi were separated, with the meninges, blood vessels and other tissues removed, cut into several small pieces with scissors and placed into a culture dish with 0.25% trypsin at 37° C for 30 min. After digestion, 1 mL of DMEM+10% FBS was added, the mixture was pipetted until there was no tissue mass, and the supernatant was aspirated into a 15 mL centrifuge tube and centrifuged at 1000 rpm for 1 minute. Then the supernatant was aspirated, added with 1 mL of DMEM+10% FBS, incubated in a petri dish at a density of 6×10^5^/mL and shaken well. The cells were cultured in a CO_2_ incubator at 37 for 4 hours and then in NEUROBASAL+2% B27+1% Glutamax. 12 days later, the cells were divided into Control group, EPO group and EPO+PHPS1 group, and then cultured under normoxia and hypoxia (5% O_2_), respectively.

### Western blotting

The tissues were lysed twice with lysis buffer, and the protein was extracted by centrifugation at 4r. After separation by SDS-PAGE, the protein was loaded (20 μ0e placed), transferred onto a PVDF membrane, blocked with 5% BSA and incubated with the primary antibodies against EPO (Abcam: ab273070, 1:5000), p-EPOR (Abcam: ab275686, 1:2000), P22 (Abcam: ab75941, 1:1000), P47 (Abcam: ab308256, 1:1000), p-SHP2 (Abcam: ab62322, 1:1000), p-TrKB (Abcam: ab229908, 1:1000), p-PI3K (Abcam: ab278545, 1:2000), p-ERK1/2 (Abcam: 184699, 1:10000), Bcl-2 (Abcam: ab194583, 1:2000), Caspase-3 (Abcam: ab184787, 1:2000), Caspase-9 (Abcam: ab184786, 1:1000), Bax (Abcam: ab289364,1:2000), and GAPDH (Abcam: ab9485, 1:2500).

After washing with TBST 3 times (5 min each time), the membrane was incubated with the corresponding secondary antibodies for 2 h and washed again. Finally, exposure, color development and result analysis were performed.

### Statistical analysis

SPSS 18.0 software was used for data analysis; Measurement data of normal distribution or approximate normal distribution were described by mean ± standard deviation (Mean ± SD); The mean of the two samples was compared by independent-samples *t*-test; The mean of two or more groups was compared by one-way analysis of variance (One-way ANOVA); Multiple comparisons were performed within the group by Student-Newman-Keuls (SNK) test; Following variable transformation and chi-square test, the rate and constituent ratio were compared in the case of heterogeneous variance; Repeated measures ANOVA was used to analyze the results of Morris water maze test; Epsilon correction was applied to asymmetrical data at each time point. P<0.05 was considered statistically significant.
